# Intra-familial phenotypic heterogeneity in a Sudanese family with *DARS2*-related leukoencephalopathy, brainstem and spinal cord involvement and lactate elevation: a case report

**DOI:** 10.1186/s12883-018-1180-7

**Published:** 2018-10-23

**Authors:** Ashraf Yahia, Liena Elsayed, Arwa Babai, Mustafa A. Salih, Sarah Misbah El-Sadig, Mutaz Amin, Mahmoud Koko, Rayan Abubakr, Razaz Idris, Shaimaa Omer M.A. Taha, Salah A. Elmalik, Alexis Brice, Ammar Eltahir Ahmed, Giovanni Stevanin

**Affiliations:** 1 Department of Biochemistry, Faculty of Medicine, University of Khartoum, Khartoum, Sudan; 2 Institute of Endemic Diseases, University of Khartoum, Khartoum, Sudan; 3 Division of Pediatric Neurology, Department of Pediatrics, College of Medicine, King Saud University, Riyadh, Saudi Arabia; 4 Department of Medicine, Faculty of Medicine, University of Khartoum, Khartoum, Sudan; 5 Department of Neurology & Epileptology, Hertie Institute for Clinical Brain Research, University of Tübingen, Tübingen, Germany; 6 Department of Radiology, Dar Al Elaj specialized hospital, Khartoum, Sudan; 7 Department of Physiology, Faculty of Medicine, University of Khartoum, Khartoum, Sudan; 8 Department of Neurology, Soba University Hospital, Khartoum, Sudan; 9 Department of Biochemistry, Faculty of Medicine, National University, Khartoum, Sudan; 10 Ecole Pratique des Hautes Etudes, EPHE, PSL Research University, Paris, France; 11 Department of Genetics, APHP, Pitié-Salpêtrière Hospital, Paris, France; 12 Institut du Cerveau et de la Moelle épinière, INSERM U1127, CNRS UMR7225, Sorbonne Universités UMR_S1127, 75013 Paris, France; 13 Department of Physiology, College of Medicine, King Saud University, Riyadh, Saudi Arabia

**Keywords:** LBSL, *DARS2*, Clinico-radiological dissociation, Intra-familial phenotypic heterogeneity, Africa

## Abstract

**Background:**

Leukoencephalopathy with brainstem and spinal cord involvement and lactate elevation (LBSL, OMIM #611105) is a genetic disease of the central nervous system characterized by lower limb spasticity, cerebellar ataxia and involvement of the dorsal column. The disease is caused by mutations in the *DARS2* gene but has never been reported in sub-Saharan Africa so far.

**Case presentation:**

Two siblings, aged 18 years and 15 years, from a consanguineous family presented with pyramidal signs and symptoms since infancy and developmental delay. Whole exome sequencing of the proband identified two compound heterozygous variants (NM_018122.4:c.1762C > G and c.563G > A) in *DARS2*. Sanger sequencing confirmed the presence of the mutations and their segregation in *trans* in both patients and in their elder sister (aged 20 years), who showed only brisk reflexes and mild lower limb spasticity. Surprisingly, in contrast to her subtle clinical presentation, the elder sister had abnormal MRI features and serum lactate levels comparable to her ill sisters.

**Conclusion:**

This report illustrates intra-familial phenotypic variation in LBSL and provides an example of a marked dissociation between the clinical and radiological phenotypes of the disease. This may have implications for the detection of mutation carriers in LBSL.

## Background

Leukoencephalopathy with brainstem and spinal cord involvement and lactate elevation (LBSL, OMIM # 611105) is a genetic disease of the central nervous system characterized by lower limb spasticity, cerebellar ataxia and involvement of the dorsal column [1]. The clinical presentation is variable both in age at onset (early childhood or adulthood) and in associated features (learning difficulty, epilepsy, mental deterioration and others) [2]. Brain magnetic resonance imaging (MRI) shows diffuse cerebral white matter changes with signal abnormalities in the dorsal column and lateral corticospinal tracts in addition to spectroscopic findings of increased lactate [1].

LBSL is an autosomal recessive disease caused by mutations in *DARS2* [3]. This gene, located on chromosome 1, has 17 exons and encodes the mitochondrial aspartyl-tRNA synthetase [3]. Defects in this gene in neurons impair the translation of mitochondrial mRNAs, leading to mitochondrial dysfunction and progressive cell loss [4]. We report here the identification of two compound heterozygous rare variants (NM_018122.4:c.1762C > G and c.563G > A) segregating in a Sudanese family with a wide clinical spectrum of LBSL and a marked dissociation between clinical and radiological phenotypes in one of the affected siblings.

## Case presentation

**Fig. 1:**
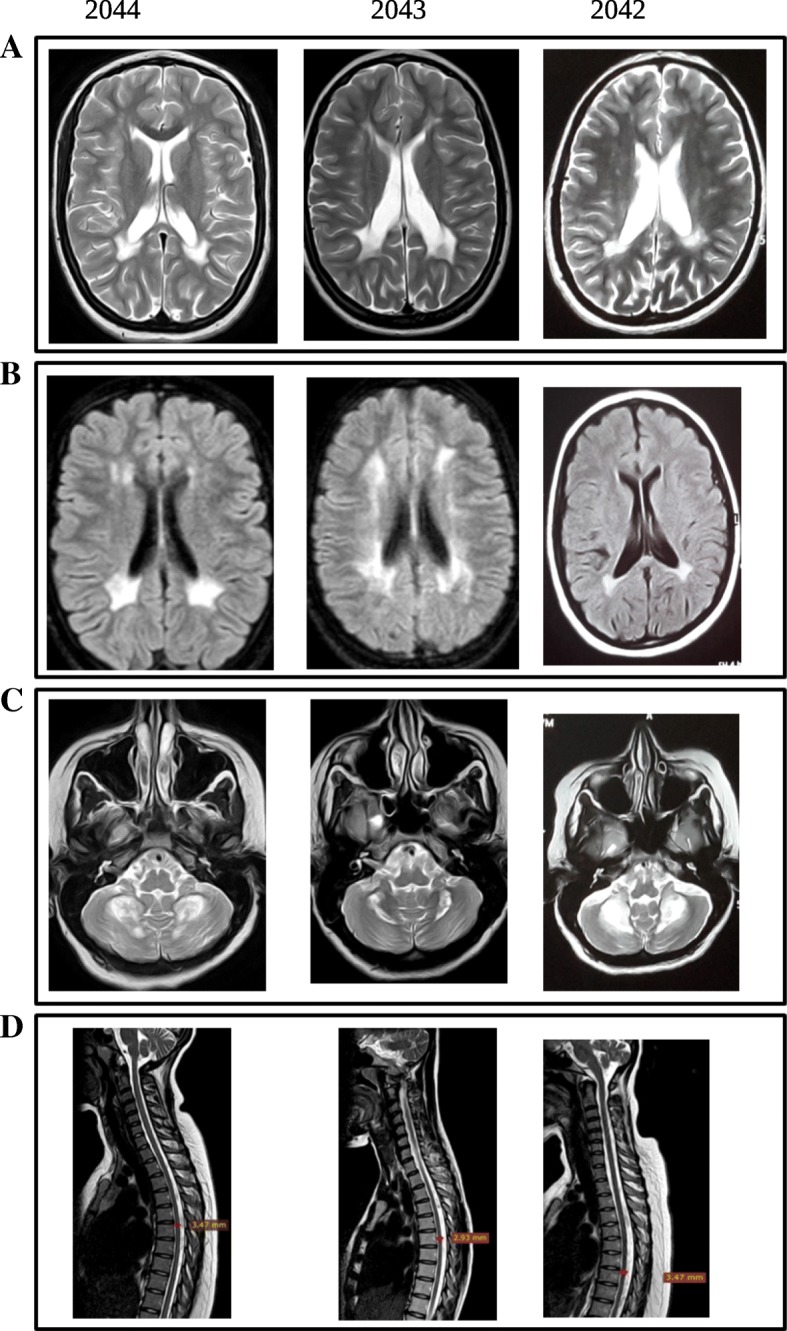
Brain and spinal cord MRIs of the three patients. MRIs of subjects 2044, 2043 and 2042 ordered from left to right. **a** and **b** Axial T2 and fluid-attenuated inversion recovery (FLAIR) sections of brain MRIs showing abnormal periventricular white matter high signal intensities in the three subjects. **c** Axial T2 MRIs through the cerebellar hemispheres demonstrate mild cerebellar atrophy in the three subjects. **d** Sagittal T2 MRIs of the spinal cord show spinal cord atrophic changes in the three subjects

Two siblings, aged 18 years and 15 years, from a Sudanese family (individuals 2043 and 2044) presented with pyramidal features since infancy (Table 1). Both patients were the outcome of uncomplicated normal vaginal deliveries and developed normally before the initial symptoms of the disease. Patient 2043 developed her symptoms from the age of 8 months after an attack of fever complicated by febrile convulsions. Initially, she manifested floppiness, which later turned into spasticity. Speech and walking were delayed (achieved at ages 3–4 and 6 years, respectively). At the age of 10 months she had three attacks of seizures, which were later controlled by carbamazepine. Spasticity progressed over time and currently the patient can only walk with support. In patient 2044, the disease started at the age of 4 months with initial floppiness followed by spasticity and delayed motor development as well, but without any history of convulsions or precipitating febrile illness. Speech developed normally. Her motor disability was analogous to that of her sister. Examination of the lower limbs showed severe spasticity, hyperreflexia and “up-going” plantar reflex in both patients. Whereas patient 2043 showed severe proximal and distal lower limb weakness, patient 2044 manifested only moderate proximal weakness. Due to the severe spasticity, heel on shin test was not applicable. Upon examination of the upper limbs, the two patients showed increased reflexes with normal tone and a normal finger-to-nose test. Ocular cerebellar signs (nystagmus, slow saccades and interrupted pursuit) were present in both patients. There were no extrapyramidal signs. Sensory nervous system and fundus examinations were normal. Both patients had normal cognitive functions and they were able to graduate from high school and attend university. Brain MRIs of patients 2043 and 2044 showed abnormal high signal intensity in the periventricular white matter and dentate nuclei bilaterally together with thinning of corpus callosum and cerebral and cerebellar atrophy (Fig. 1). Spinal MRI showed signal changes with dorsal spinal cord atrophic changes in both these affected sisters.

**Table 1:** Clinical characteristics of the described genetically affected siblings

Patient ID	2042	2043	2044
Gender	Female	Female	Female
Age at examination	20 years	18 years	15 years
Age at onset	–	8 months	4 months
Initial sign / symptom	Hypertonia and hyperreflexia detected during the sampling session	Floppiness	Floppiness
Delayed motor development	–	+	+
Delayed speech	–	+	–
Epilepsy	–	+	–
Cognitive impairment	–	–	–
Degree of motor disability	No functional handicap but signs at examination	Walk with support / unable to run	Walk with support / unable to run
Muscle wasting (UL & LL)	–	–	–
UL hypertonia	–	–	–
UL motor deficit	–	–	–
UL hyperreflexia	+	+	+
LL hypertonia	+	+	+
LL motor deficit	–	Severe	Moderate
LL hyperreflexia	+	+	+
Sensory impairment	–	–	–
Dysarthria	–	+	+
Ocular cerebellar signs	–	+	+
Dysmetria	–	–	–
Optic atrophy	–	–	–
Clinical summary	Pyramidal features	Pyramidal features, seizures, delayed speech, ocular cerebellar signs and dysarthria	Pyramidal features, ocular cerebellar signs and dysarthria
MRI changes in brain and spinal cord	+	+	+
Serum lactate level in mmol/L (reference range 0.5–2.2 mmol/L)	6.13	6.8	5.97

*UL* upper limb, *LL* lower limb, − absent, + present

Four first-degree relatives were assessed for inclusion as controls in the genetic analysis: the parents (2040 and 2041), an elder sister (2042), and a younger sister (2045). They underwent routine clinical examination to rule out the possibility of subtle abnormalities. The examination was normal in the parents and the younger sister. The elder sister showed brisk reflexes in both upper and lower limbs and mild spasticity in the lower limbs but no other clinically detectable abnormality (Table 1).

**Fig. 2:**
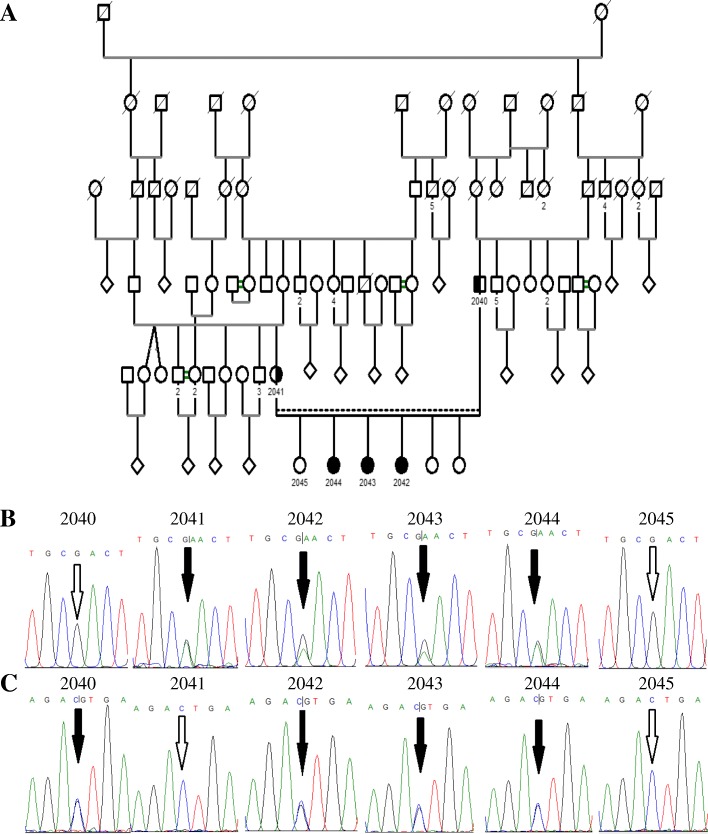
Pedigree and segregation analysis. Segregation analysis shows compound heterozygous pattern of disease inheritance. **a** Family pedigree. **b** and **c** Electropherograms showing the segregation pattern of c.563G > A and c.1762C > G (black arrows), in (**b**) and (**c**), respectively; in subjects 2040, 2041 (parents), 2045 (healthy sister), 2042, 2043 and 2044 (patients). White arrows point to wild type variants at the genomic positions of interest. The variant c.563G > A is inherited from the mother (2041) while the variant c.1762C > G is inherited from the father

DNA was extracted from saliva samples. Whole exome sequencing was performed in the proband (patient 2043) and revealed two compound heterozygous rare variants in the *DARS2* gene (NM_018122.4: c.1762C > G and NM_018122.4: c.563G > A; p.(Leu588Val) and p.(Arg188Gln), respectively). The variants were predicted as pathogenic using three pathogenicity prediction tools (SIFT [5], PolyPhen-2 [6] and MutationTaster [7]). The variant c.1762C > G (rs972404343) was reported as “likely pathogenic” in the ClinVar database [8]; it was absent from both the ExAC and gnomAD databases [9]. The second variant, c.563G > A (rs182811621), was not reported in the ClinVar database but had very low allele frequencies in the ExAC and gnomAD databases (0.00001 and 0.000004, respectively) [9]. Sanger sequencing (Fig. 2) identified the father and mother as heterozygous carriers of the single variant c.1762C > G and c.563G > A, respectively, and validated the presence of both variants in patients 2043 and 2044. Additionally, subject 2042, who had only mild signs on examination, was found to harbor both variants.

To further investigate the genotype-phenotype correlation as well as the association between the clinical and radiological findings, brain and spinal MRIs were obtained for patient 2042. Interestingly, she presented similar features to her affected siblings in the form of periventricular white matter, dentate nuclei, medulla oblongata and cervical spinal cord areas of signal changes and cerebral and spinal cord atrophic changes (Fig. 1). Biochemical investigations showed an elevated serum lactate level (reference range: 0.5–2.2 mmol/L) in patients 2043 and 2044 (6.8 mmol/L and 5.97 mmol/L, respectively) and in patient 2042 (6.13 mmol/L) as well. Currently, the younger patients are on regular physiotherapy. No further medical intervention has been undertaken in the minimally affected elder sister.

## Discussion and conclusion

Compound heterozygous mutations are implicated in the majority of LBSL cases [10]. There is a wide variety in the clinical presentation of patients [10]. In addition, there is no apparent genotype-phenotype correlation nor is there a correlation between the degree of mitochondrial aspartyl-tRNA synthetase dysfunction and disease severity [11]. In this report, we showed that patients in the same family with the same compound heterozygous mutations can vary in their clinical presentations from an apparently healthy individual (with findings limited to brisk reflexes incidentally recognized at age 20 years) to disabled patients. Additionally, we highlighted the marked dissociation that can occur between clinical phenotype and MRI findings in LBSL. To the best of our knowledge, such a dissociation has only previously been reported in two sisters diagnosed with asymptomatic LBSL due to compound heterozygous mutations in *DARS2* [12]. On the other hand, the dissociation between clinical and radiological phenotypes has been reported in other forms of leukodystrophies [13, 14]. However, the biological basis of this dissociation has yet to be unraveled. In our opinion biochemical, radiological or genetic screening of healthy siblings of LBSL patients could be of value to rule out the presence of the disease. Detecting asymptomatic/minimally symptomatic patients is of value in premarital counseling, especially in countries where consanguineous marriage is common. Studies that are more comprehensive could determine the sensitivity, specificity and cost-effectiveness of such screening methods. Our study is the first to report cases of LBSL from sub-Saharan Africa. Nevertheless, functional studies are still needed to confirm the pathogenicity of the reported variants.

In conclusion, LBSL can show marked phenotypic variability even within the same family. This variable expressivity may complicate the detection of the causative variants in the context of genetic counseling.

## Abbreviations

FLAIR: Fluid-attenuated inversion recovery
LBSL: Leukoencephalopathy, brainstem and spinal cord involvement and lactate elevation
MRI: Magnetic resonance imaging
OMIM: Online mendelian inheritance in man
